# Epidemiological Comparative Study on Contact Sensitisations in Woodworkers With Occupational Dermatitis: Patch Test Data of the Information Network of Departments of Dermatology, 1999 to 2023

**DOI:** 10.1111/cod.70041

**Published:** 2025-11-12

**Authors:** Luisa Heizmann, Steffen Schubert, Andrea Bauer, Detlef Becker, Knut Brockow, Heinrich Dickel, Birger Kränke, Claudia Lang, Eva Oppel, Nicola Wagner, Elke Weisshaar, David Wilfinger, Richard Brans, for the IVDK, Ulrike Beiteke, Joachim Dissemond, Timo Buhl, Knut Schäkel, Claudia Pföhler, Guido Heine, Margitta Worm, Burkhard Kreft, Jörg Tittelbach, Sebastian Volc, Andreas Recke, Jana Witte, Wolfgang Pfützner, Brigitte Coras‐Stepanek, Christoph Skudlik, Rüdiger Panzer, Jens Malte Baron, Franziska Grän, Vera Baur, Jan Nicolay, Beatrice Schell, Axel Trautmann, Karin Hartmann, Christiane Szliska, Johannes Weiß, Gertrud Grundmann, Michael Jünger, Mathias Sulk, Annice Heratizadeh, Juliane Rieker‐Schwienbacher, Dieter Vieluf, Ralf Gutzmer, Dagmar Simon, Manigé Fartasch, Susann Forkel, Stefan Nestoris, Dirk Mechtel, Claudia Schröder‐Kraft, Harald Löffler, Michal Gina, André Koch, Ulrike Raap, Zita Manjaly‐Thomas, Welf Prager, Kerstin Strom, Irena Angelova‐Fischer, Susanne Buder, Philipp Spring

**Affiliations:** ^1^ Institute for Interdisciplinary Dermatologic Prevention and Rehabilitation (iDerm) at the Osnabrück University Osnabrück Germany; ^2^ Information Network of Departments of Dermatology (IVDK) Institute at the Georg‐August University of Göttingen Göttingen Germany; ^3^ Department of Dermatology University Allergy Center, University Hospital Carl Gustav Carus, Technical University Dresden Dresden Germany; ^4^ Department of Dermatology University of Mainz Mainz Germany; ^5^ Department of Dermatology and Allergy Biederstein School of Medicine and Health, Technical University of Munich (TUM) Munich Germany; ^6^ Department of Dermatology, Venereology and Allergology St. Josef Hospital, University Medical Center, Ruhr University Bochum Bochum Germany; ^7^ Department of Dermatology and Venereology Medical University of Graz Graz Austria; ^8^ Department of Dermatology University Hospital Zurich Zurich Switzerland; ^9^ Immunologie‐Zentrum Zürich Zurich Switzerland; ^10^ Department of Dermatology and Allergology Ludwig‐Maximilians‐University Munich Germany; ^11^ Department of Dermatology Uniklinikum Erlangen, Friedrich‐Alexander‐University of Erlangen‐Nürnberg (FAU) Erlangen Germany; ^12^ Division of Occupational Dermatology, Department of Dermatology University Hospital Heidelberg, Ruprecht Karls University Heidelberg Heidelberg Germany; ^13^ Department of Occupational Diseases and Occupational Medicine AUVA Rehabilitation Clinic Tobelbad Tobelbad Austria; ^14^ Department of Dermatology Environmental Medicine and Health Theory, Osnabrück University Osnabrück Germany

**Keywords:** airborne, allergic contact dermatitis, colophonium, epoxy resin, hand dermatitis, occupational, patch test, woodworkers

## Abstract

**Background:**

Woodworkers (WW) are exposed to a wide range of occupational hazards and potent allergens.

**Objectives:**

To describe the contact sensitisation pattern of WW with occupational dermatitis (OD).

**Methods:**

In a retrospective study, patch test and clinical data collected by the Network of Departments of Dermatology (IVDK) from 1999 to 2023 were analysed. Data of 471 WW with OD (89.6% male) were compared with data of 558 WW without OD and 39 759 patients from other occupational groups (non‐WW) with OD.

**Results:**

Allergic contact dermatitis (32.5%) was the most frequent diagnosis among WW with OD. Hands (63.5%) were predominantly involved. Face dermatitis (8.5%) and dust exposure (36.6%) as contributing factors were significantly more common than in non‐WW with OD. Sensitisations to epoxy resin (5.5%) were significantly more frequent in male WW with OD than in male WW without OD. Other frequent sensitisations included those to *Myroxylon pereirae* (7.4%), propolis (5.6%), and colophonium (5.3%). Sensitisations to chromium, cobalt, thiuram mix, fragrance mix II and compositae mix II were significantly less frequent than in male non‐WW patients with OD.

**Conclusions:**

Allergic contact dermatitis is frequent in WW with OD. Important allergens include resins and plant‐derived substances. Airborne OD may interfere with prevention efforts.

## Graphical abstract

The data demonstrates that allergic contact dermatitis is common in WW with OD. The most important allergens include resins (including epoxy resin) and plant‐derived substances (including colophonium). The high share of WW with allergic contact dermatitis and face dermatitis hampers preventive efforts.

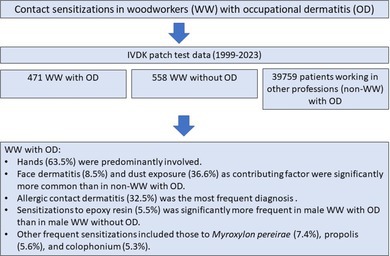

## 1 Introduction

The professional group of woodworkers, which is often included among construction workers, encompasses a wide range of different craft occupations, such as carpenters, joiners, veneers, loggers, pattern makers, wood turners, wood carvers, braid makers and basketry makers [1, 2]. In this manuscript they are all collectively referred to as woodworkers (WW). Professionally, WW are exposed to various irritative and/or allergenic substances, such as sawdust, paints, varnishes, wood stains, adhesives, spray foams, solvents, and insulating materials [2, 3]. Moreover, the hands are exposed to mechanical strain, for example, by carrying rough‐surfaced, heavy loads and the use of hand‐held tools, including saws, drills, grinders, hammers and screw drivers. Due to outdoor work, some WW are additionally exposed to the varying weather conditions, which, especially during the fall and winter months, results in skin strain due to coldness and wetness [2]. All these skin hazards contribute to an increased risk of developing occupational dermatitis (OD) in WW, manifesting as irritant and/or allergic contact dermatitis particularly on the hands. The incidence per 10 000 person‐years of occupational skin diseases in WW has been calculated in a few studies and was estimated to be 2.6 (95% confidence interval (CI) 1.9–3.4) in wood processors in Northern Bavaria (1990–1999) [4] and 1.98 (95% CI 1.4–2.7) in carpenters and joiners in Finland (2005–2016) [5].

Individuals affected by OD not only experience distressing symptoms, such as itch or pain, but are also often emotionally burdened by the discomfort and limitations the skin condition imposes on both their professional and personal lives resulting in an impaired health‐related quality of life [6, 7]. Affected individuals are often incapable of working due to OD, frequently over extended periods [8]. They face an increased risk of unemployment or must cope with reduced income due to for example, the necessity of retraining [9]. This underlines the importance of preventive measures.

This study aims to identify the pattern of contact sensitisations among WW with OD to facilitate the implementation of targeted preventive measures for this occupational group. Furthermore, the findings of this study will enable the refinement of patch testing recommendations for WW.

## 2 Methods

### 2.1 Patients

The Information Network of Departments of Dermatology (IVDK), is a network of departments across Germany, Switzerland and Austria. Its structure and routine operating procedures are described in detail elsewhere [10]. Briefly, patients' histories, clinical data, and patch test results are recorded in local databases in the participating centres and, after pseudonymisation, transmitted to the IVDK central office at the University of Göttingen, Germany. This also includes the selection of at least one and up to 3 allergen sources suspected to be causative for the skin lesions prior to patch testing. Data are subjected to standardised quality control, added to the central IVDK database, and analysed according to international standards [11]. The study was conducted in accordance with the ethical principles for medical research involving human subjects (WMA declaration of Helsinki) and was approved by the ethics committee of the University Medical Center Göttingen (18/12/24). Written informed consent was obtained from all patients. The following data analysis utilises data from the IVDK, collected by 62 participating departments of dermatology in the period from 1999 to 2023. The study group consisted of all WW with OD whose data were collected in the given period of time. The comparison groups consisted of male WW without OD and male patients from other occupational groups (non‐WW) with OD patch tested in the same period of time. The WW without OD had non‐occupational skin disorders (mainly dermatitis) which led to patch testing to confirm or exclude allergic contact dermatitis. WW with missing or unknown information on OD status were excluded from further analyses.

### 2.2 Patch Testing

Patch testing and evaluation of reactions were conducted according to the guidelines established by the German Contact Dermatitis Research Group (DKG) [12, 13]. Patch test readings were performed at least twice, most often on day (D) 2 and 3. This data analysis primarily considered patch test reactions on D3. In case a D3 reading was unavailable, the patch test reactions on D4 were included instead. Later readings were not routinely performed. Readings classified as +, ++, or +++, indicating reactions with erythema, infiltration, papules, and/or (coalescing) vesicles, were categorised as positive. Patch test preparations were purchased from Almirall Hermal, Reinbek, Germany (until 2013), SmartPractice Europe, Greven, Germany (from 2014 on), and in exceptional cases from Chemotechnique Diagnostics, Vellinge, Sweden. The composition of the test series as well as concentrations of single preparations changed during the 24‐year study period, resulting in varying numbers of patients tested with each single test allergen. Patch test results involving patients' own materials were excluded from the analysis.

### 2.3 Data Analysis

Data analysis was conducted in the IVDK central office with the SAS 9.4 software package (SAS Institute, Cary, NC, USA). Consideration for comparing the patch test results of WW with OD to those of the two comparison groups was primarily given to the DKG baseline series as it provides a representative comparative dataset from all three groups. The vast majority of WW with OD were male. Therefore, and to avoid a sex bias, the comparative analyses of sensitisations to the DKG baseline series allergens between WW with OD and the two comparison groups were only done in males. Reaction rates were standardised to age (50% < 40 years, 50% ≥ 40 years) using previously published methods [14, 15]. Significant differences in anamnestic items and patch test results were concluded from non‐overlapping 95% confidence intervals (CIs) [11]. Additionally, a multivariate logistic regression analysis was performed in mutual control for three dichotomized explanatory variables, namely positive patch test reactions to epoxy resin, colophonium, and 4,4′‐diaminodiphenylmethane, and the diagnosis of face dermatitis among WW with OD as the target variable. Model convergence was achieved within the predefined maximum number of iterations. Observations were independent, and no complete separation was observed. The model shows no signs of multicollinearity by checking the events‐per‐variable ratio (EPV), variance inflation factors and pairwise Phi coefficients between explanatory variables.

## 3 Results

### 3.1 Patients Characteristics

Between 1999 and 2023, a total of 252 683 patients underwent patch testing in terms of 262 655 consultations within the IVDK network. Among these were 1245 WW (with and without OD) and 39 759 non‐WW with OD. Among the 1245 WW, 37.8% (*n* = 471) were diagnosed with OD while 44.8% (*n* = 558) did not have OD (Table 1). In the remaining patch‐tested WW, information on OD was missing or unknown. These were excluded from further analyses.

**TABLE 1:** Population characteristics of woodworkers (WW) with occupational dermatitis (OD), WW without OD and all patients not working as woodworkers (non‐WW) with OD.

Population characteristics		WW with OD (*n* = 471)		WW without OD (*n* = 558)		Non‐WW with OD (*n* = 39 759)	
		*n*	% [95% CI]	*n*	% [95 CI]	*n*	% [95 CI]
M	Male	422	89.6 [86.5–92.2]	481	86.2 [83.1–89.0]	19 091	48.0 [47.5–48.5]
O	Occupational dermatitis	471	100.0^a^	0	0^a^	39 759	100.0^a^
A	Atopic dermatitis (past or present)	125	26.5 [22.8–30.7]	116	20.8 [17.6–24.4]	12 816	32.2 [31.8–32.7]
H	Hand dermatitis^b^	299	63.5 [59.0–67.8]	148	26.5 [22.9–30.4]	32 297	81.2 [80.8–81.6]
L	Leg dermatitis^b^	7	1.5 [0.6–3.0]	50	9.0 [6.7–11.6]	279	0.7 [0.6–0.8]
F	Face dermatitis^b^	40	8.5 [6.1–11.4]	67	12.0 [9.4–15.0]	2244	5.6 [5.4–5.9]
A	Age ≥ 40	229	48.6 [44.0–53.2]	328	58.8 [54.6–62.9]	21 565	54.2 [53.7–54.7]
	Diagnoses^b^						
	Allergic contact dermatitis	153	32.5 [28.3–36.9]	95	17.0 [14.0–20.4]	12 376	31.1 [30.7–31.6]
	Irritant contact dermatitis	89	18.9 [15.5–22.7]	30	5.4 [3.7–7.6]	9950	25.9 [24.6–25.5]
	Atopic dermatitis	59	12.5 [9.7–15.9]	63	11.3 [8.8–14.2]	4352	10.9 [10.6–11.3]
	Hyperkeratotic dermatitis	43	9.1 [6.7–12.1]	24	4.3 [2.8–6.3]	2264	5.7 [5.5–5.9]
	Contributing co‐factors^c^	325	69.0 [64.6–73.2]	152	27.2 [23.6–31.1]	29 350	73.8 [73.4–74.3]
	Dusts	119	36.6 [31.4–42.1]	35	23.0 [16.6–30.5]	1505	5.1 [4.9–5.4]
	Occlusive environment	62	19.1 [15.0–23.8]	30	19.7 [13.7–27.0)	11 459	39.0 [38.5–39.6]
	Mechanical strain	48	14.8 [11.1–19.1]	20	13.2 [8.2–19.6]	1936	6.6 [6.3–6.9]
	Wetness	21	6.5 [4.0–9.7]	8	5.3 [2.3–10.1]	8776	29.9 [29.4–30.4]

Abbreviations: 95% CI, 95% confidence interval; OD, occupational dermatitis; WW, woodworkers.

^a^ By group definition.

^b^ Selected main diagnoses.

^c^ Selection of the four most common cofactors in WW with OD, the proportions of single co‐factors refer to all those with contributing co‐factors.

In the present data set, the group of WW with OD primarily consisted of carpenters and joiners (*n* = 246, 52.2%). A full list of occupations is presented in Table 2. Similarly, the majority of WW without OD were carpenters and joiners (*n* = 269, 48.2%) (Table S1). Non‐WW with OD belonged to various occupational groups (Table S2).

**TABLE 2:** Occupations of woodworkers (WW) with occupational dermatitis (OD).

Occupation	*n*	%
Carpenter (construction, furniture, coffin), joiner	246	52.2
Roofer, prefabricated wood assembler	82	17.4
Wood preparer, sawmill worker, veneer producer, and so forth.	55	11.7
Carpenter, woodworker, and so forth (unspecified)	27	5.7
Wood surface finisher, stainer, polisher, veneerer	21	4.5
Model carpenter, mould carpenter	19	4
Wood shaper, wood turner, wood carver	17	3.6
Wooden goods maker (brushes, toys, etc.)	3	0.6
Basket and wickerwork maker	1	0.2

The proportions of characteristics of WW with OD compared with WW without OD as well as with non‐WW with OD are presented in Tables 1 and 3. The patch tested WW consisted predominantly of men (WW with OD: 89.6%, 95% CI 86.5–92.2; WW without OD: 86.2%, 95% CI 83.1–89.0) while non‐WW with OD were significantly more often women (male: 48.0%, 95% CI 47.5–48.5).

**TABLE 3:** The five most common main suspected allergen sources (up to three per patient could be indicated) prior to patch testing of woodworkers (WW) with occupational dermatitis (OD), WW without OD and all patients not working as woodworkers (non‐WW) with OD.

Suspected allergen sources	WW with OD		Suspected allergen sources	WW without OD		Suspected allergen sources	Non‐WW with OD	
	*n*	% (95% CI)		*n*	% (95% CI)		*n*	% (95% CI)
Woods	233	49.5 [44.9–54.1]	Leave‐on cosmetics	138	24.7 [21.2–28.5]	Gloves (various materials)	11 799	29.7 [29.2–30.1]
Adhesives/glues	116	24.6 [20.8–28.8]	Woods	136	24.4 [20.9–28.2]	Disinfectants	9712	24.4 [24.0–24.9]
Gloves (various materials)	83	17.6 [14.3–21.4]	External drugs	83	14.9 [12.0–18.1]	Leave‐on cosmetics	7583	19.1 [18.7–19.5]
Paints and lacquers	80	17.0 [13.7–20.7]	Glues	61	10.9 [8.5–13.8]	Rubber	5973	14.9 [14.6–15.3]
Building materials	61	13.0 [10.1–16.3]	Systemic drugs	48	8.6 [6.4–11.2]	Water‐based cutting fluids	5230	13.2 [12.8–13.5]

Abbreviations: 95% CI, 95% confidence interval; OD, occupational dermatitis; WW, woodworkers.

The mean age of WW with OD was 39.1 years (median: 39; 25% percentile: 28; 75% percentile: 50) and 48.6% of them were aged ≥ 40 years which was significantly lower than in both comparison groups. Past or present atopic dermatitis was significantly less frequent in WW with OD (26.5%, 95% CI 22.8–30.7) than in non‐WW with OD (32.2%, 95% CI 17.6–24.4). The majority of WW with OD had hand dermatitis (63.5%, 95% CI 59.0–67.8) which was significantly increased compared to WW without OD (26.4%, 95% CI 22.9–30.4), but significantly less common than in non‐WW with OD (81.2%, 95% CI 80.8–81.6). In addition, 8.5% of WW with OD (95% CI 6.1–11.4) had face dermatitis which was significantly increased compared to non‐WW with OD (5.6%, 95% CI 5.4–5.9). In WW without OD, face dermatitis was also frequent (12.0%, 95% CI 9.4–15.0). Leg dermatitis was significantly increased in WW without OD (9.0%, 95% CI 6.7–11.6) compared to the other two groups. In WW with OD, the main suspected allergen sources prior to patch testing were woods (49.5%), adhesives/glues (24.6%), and gloves made from various materials (17.6%), closely followed by paints and lacquers (17.0%) and building materials (13.0%) (Table 3). This differed extensively from the two comparison groups. In non‐WW with OD, gloves (29.7%) were the main suspected allergen source.

In the group of WW with OD, the final main diagnosis of allergic contact dermatitis (32.5%, 95% CI 28.3–36.9) was significantly more common than irritant chronic contact dermatitis (18.9%, 95% CI 15.5–22.7) (Table 1). Other frequent main diagnoses were atopic dermatitis (12.5%, 95% CI 9.7–15.9) and hyperkeratotic eczema (9.1%, 95% CI 6.7–12.1). A comparable share of non‐WW with OD suffered from allergic contact dermatitis (31.1%, 95% CI 30.7–31.6), but significantly less WW without OD had this main diagnosis (17.0%, 95% CI 14.0–20.4). A main diagnosis of hyperkeratotic dermatitis was significantly more common in WW with OD (9.1%, 95% CI 6.7–12.1) than in those without OD (4.3%, 95% CI 2.8–6.3) and in non‐WW with OD (5.7%, 95% CI 5.5–5.9).

Contributing co‐factors were significantly less frequently identified in WW with OD (69.0%, 95% CI 64.6–73.2) than in non‐WW with OD (73.8%, 95% CI 73.4–74.3), but significantly more frequently than in WW without OD (27.2%, 95% CI 23.6–31.1). The four most commonly attributed co‐factors in WW with OD were dusts (36.6%, 95% CI 31.4–42.1), occlusive environment (19.1%, 95% CI 15.0–23.8), mechanical strain (14.8%, 95% CI 11.1–19.1), and wetness (6.5%, 95% CI 4.0–9.7). In non‐WW with OD, dusts (5.1%, 95% CI 4.9–5.4) and mechanical strain (6.6%, 95% CI 6.3–6.9) were significantly less frequently, and occlusive environment (39.0%, 95% CI 38.5‐39.6) and wetness (29.9%, 95% CI 29.4‐30.4) significantly more frequently identified co‐factors than in WW with OD.

### 3.2 Patch Test Results

The DKG baseline series was tested in 91.7% WW with OD, in 90.2% of WW without OD and in 91.9% of non‐WW with OD. The reaction frequencies standardised to age (50% < 40 years, 50% ≥ 40 years) and 95% CI of the males in the three patient groups are presented in Table 4.

**TABLE 4:** Age‐standardised (50% men < 40 years, 50% men ≥ 40 years) proportions of positive patch test reactions, supplemented with 95% confidence intervals, to allergens of the DKG baseline series in male woodworkers (WW) with occupational dermatitis (OD) compared to male WW without OD and all other male patients with OD not working as WW (non‐WW).

Test substance	Male WW with OD				Male WW without OD			Male non‐WW with OD		
	Conc.	Tested *n*	Positive *n*	Standard pos. % [95% CI]	Tested *n*	Positive *n*	Standard pos. % [95% CI]	Tested *n*	Positive *n*	Standard pos. % [95% CI]
p‐Phenylenediamine	0.3%	26	3	11.9 [0.0–25.0]	14	1	25.0 [0.0–74.0]	1411	39	2.8 [1.9–3.7]
*Myroxylon pereirae*	25%	381	28	7.4 [4.8–10.0]	425	17	3.6 [1.9–5.3]	17 092	869	4.8 [4.4–5.1]
Methylisothiazolinone (aq.)	0.05%	231	13	5.9 [2.8–9.0]	223	3	1.4 [0.0–3.0]	11 072	675	5.8 [5.3–6.2]
MDBGN	0.3%	136	8	5.6 [1.8–9.4]	185	4	2.0 [0.0–4.0]	7387	407	5.2 [4.7–5.7]
Propolis	10%	376	21	**5.6 [3.3–7.9]** ^a^	409	12	2.5 [1.1–3.9]	16 863	435	2.5 [2.3–2.7]
Epoxy resin	1%	380	21	**5.5 [3.2–7.8]** ^b^	422	5	1.3 [0.1–2.4]	16 757	905	5.3 [5.0–5.7]
Colophonium (Rosin)	20%	380	20	5.3 [3.0–7.6]	424	8	1.9 [0.6–3.2]	17 047	794	4.5 [4.2–4.8]
Potassium dichromate	0.5%	381	17	**4.5 [2.4–6.6]** ^c^	424	10	2.1 [0.8–3.3]	17 025	1240	7.0 [6.7–7.4]
Nickel (II)‐sulfate	5%	378	16	4.3 [2.2–6.3]	425	25	6.0 [3.7–8.3]	16 993	1037	6.1 [5.8–6.5]
Fragrance mix I	8%	372	15	4.1 [2.1–6.1]	424	29	6.6 [4.2–9.0]	16 881	880	5.0 [4.7–5.3]
MCI/MI (aq.)	100 ppm	378	13	3.5 [1.6–5.3]	425	9	2.1 [0.7–3.4]	17 015	817	4.6 [4.3–5.0]
Cobalt (II)‐chloride	1%	380	13	**3.5 [1.6–5.3]** ^c^	424	13	2.9 [1.3–4.6]	17 056	992	5.8 [5.4–6.2]
p‐Phenylenediamine	1%	135	4	3.3 [0.0–6.7]	192	8	4.2 [1.3–7.0]	5901	320	5.3 [4.7–5.9]
Compositae mix	6%	76	3	2.9 [0.0–6.1]	85	1	1.2 [0.0–3.4]	3349	77	2.3 [1.8–2.8]
Oil of turpentine	10%	377	9	2.4 [0.8–4.0]	424	6	1.4 [0.3–2.5]	16 964	246	1.3 [1.2–1.5]
PTBFR	1%	258	5	1.9 [0.2–3.6]	297	2	0.8 [0.0–1.8]	11 016	76	0.7 [0.5–0.8]
Lanolin alcohols	30%	379	6	1.6 [0.3–2.8]	424	9	1.9 [0.6–3.1]	17 096	249	1.4 [1.2–1.6]
Mercaptobenzothiazole	2%	340	5	1.5 [0.2–2.7]	358	4	1.3 [0.0–2.6]	15 352	238	1.5 [1.3–1.7]
Zinc diethyldithiocarbamate	1%	346	5	1.5 [0.2–2.7]	404	1	0.3 [0.0–0.9]	16 492	165	1.0 [0.8–1.1]
Mercapto mix (CBS, MBTS, MOR)	1%	359	5	1.4 [0.2–2.6]	419	3	0.8 [0.0–1.7]	16 172	247	1.5 [1.3–1.7]
IPPD	0.1%	379	5	1.3 [0.2–2.5]	424	0	0.0 [0.0–0.7]	17 082	307	1.8 [1.6–2.0]
MDBGN	0.2%	172	2	1.2 [0.0–2.8]	160	4	2.7 [0.0–5.5]	7629	237	2.9 [2.5–3.3]
Thiuram mix	1%	377	4	**1.1 [0.0–2.1]** ^c^	425	5	1.2 [0.1–2.2]	17 034	828	4.7 [4.4–5.0]
Iodopropynylbutyl carbamate	0.2%	377	4	1.1 [0.0–2.1]	425	5	1.2 [0.1–2.2]	13 412	225	1.6 [1.4–1.8]
HICC	5%	229	2	0.9 [0.0–2.1]	217	3	1.0 [0.0–2.2]	15 849	225	1.3 [1.1–1.5]
Bronopol	0.5%	246	2	0.9 [0.0–2.1]	221	5	1.7 [0.2–3.1]	14 850	150	0.9 [0.8–1.1]
Formaldehyde (aq.)	1%	382	3	0.8 [0.0–1.7]	424	4	1.1 [0.0–2.2]	17 106	285	1.6 [1.4–1.8]
Ylang ylang oil	10%	382	3	0.8 [0.0–1.7]	424	4	1.1 [0.0–2.2]	10 332	174	1.6 [1.4–1.9]
Paraben mix	16%	361	2	0.6 [0.0–1.3]	413	8	2.0 [0.6–3.4]	16 513	175	1.1 [0.9–1.2]
Cetylstearyl alcohol	20%	363	2	0.6 [0.0–1.3]	413	8	2.0 [0.6–3.4]	16 529	61	0.4 [0.3–0.4]
Sorbitan sesquioleate	20%	200	1	0.5 [0.0–1.4]	187	5	2.4 [0.3–4.6]	9012	62	0.7 [0.5–0.8]
Jasmine absolute	5%	361	2	0.6 [0.0–1.3]	413	8	2.0 [0.6–3.4]	10 199	97	0.9 [0.7–1.1]
Jasmine absolute	2%	363	2	0.6 [0.0–1.3]	413	8	2.0 [0.6–3.4]	477	1	0.3 [0.0–1.0]
Fragrance mix II	14%	195	1	**0.5 [0.0–1.3]** ^c^	180	3	1.2 [0.0–2.5]	13 860	442	2.9 [2.7–3.2]
Sandalwood oil	10%	200	1	0.5 [0.0–1.4]	187	5	2.4 [0.3–4.6]	10 815	83	0.8 [0.6–0.9]
Compositae Mix II	5%	318	0	**0.0 [0.0–0.9]** ^c^	346	3	0.7 [0.0–1.6]	8761	122	1.3 [1.0–1.5]
Compositae Mix	5%	26	0	0.0 [0.0–10.9]	30	0	0.0 [0.0–9.5]	4024	69	1.6 [1.3–2.0]
Mercaptobenzothiazole	1%	26	0	0.0 [0.0–10.9]	30	0	0.0 [0.0–9.5]	1376	22	1.5 [0.9–2.1]

*Note*: Vehicle is petrolatum unless aqua (aq.) is specified. Allergens with significantly different reaction frequencies in male WW with OD compared to at least one of the two comparison groups are highlighted in bold.

Abbreviations: 95% CI, 95% confidence interval; aq., aqua; CBS, N‐cyclohexyl‐2‐benzothiazylsulfenamide; conc., concentration; DKG, German Contact Dermatitis Research Group; HICC, hydroxyisohexyl 3‐cyclohexene carboxaldehyde; IPPD, N‐isopropyl‐N‐phenyl‐p‐phenylenediamine; MBT, 2‐mercaptobenzothiazole; MBTS, dibenzothiazyl disulfide; MCI, methylchloroisothiazolinone; MDBGN, methyldibromoglutaronitril; OD, occupational dermatitis; pos., positive; PTBFR, p‐tert.‐butylphenol formaldehyde resin; WW, woodworkers.

^a^ Significantly higher reaction frequency in male WW with OD compared to male non‐WW with OD.

^b^ Significantly higher reaction frequency in male WW with OD compared to male WW without OD.

^c^ Significantly lower reaction frequencies in male WW with OD compared to male non‐WW with OD.

The only sensitisation within the DKG baseline series that was significantly more common among male WW with OD compared to male WW without OD was to epoxy resin (WW with OD: 5.5%, 95% CI 3.2–7.8 vs. WW without OD: 1.3%, 95% CI 0.1–2.4). In contrast, the sensitisation frequency to epoxy resin in male non‐WW with OD (non‐WW with OD: 5.3%, 95% CI 5.0–5.7) was similar to that of male WW with OD. The only significantly more frequent sensitisation to DKG baseline series allergens in male WW with OD compared to male non‐WW with OD was to propolis (WW with OD: 5.6%, 95% CI 3.6–8.2 vs. non‐WW with OD: 2.5%, 95% CI 2.3–2.7). Significantly less common in male WW with OD compared to male non‐WW with OD were sensitisations to potassium dichromate (WW with OD: 4.5%, 95% CI 2.4–6.6, non‐WW with OD: 7.0%, 95% CI 6.7–7.4), cobalt (II)‐chloride (WW with OD: 3.5%, 95% CI 1.6–5.3, non‐WW with OD: 5.8%, 95% CI 5.4–6.2), thiuram mix (WW with OD: 1.1%, 95% CI 0.0–2.1, non‐WW with OD: 4.7%, 95% CI 4.4–5.0), fragrance mix II (WW with OD: 0.5%, 95% CI 0.0–1.3, non‐WW with OD: 2.9%, 95% CI 2.7–3.2), and compositae mix II (WW with OD: 0%, 95% CI 0.0–0.9, non‐WW with OD: 1.3%, 95% CI 1.0–1.5).

Other very frequent sensitisers of the DKG baseline series in male WW with OD were *Myroxylon pereirae* (6.9%, 95% CI 4.7–9.8), methylisothiazolinone (5.9%, 95% CI 2.8–9.0), and methyldibromo glutaronitrile (5.6%, 95% CI 1.8–9.4), without, however, any significant differences to the other two groups. In addition, sensitisation to colophonium (5.3%, 95% CI 3.0–7.6) was much more frequent in male WW with OD compared to male WW without OD (1.9%, 95% CI 0.6–3.2), even though again not reaching significance. Sensitisations to p‐phenylenediamine (PPD) were also frequent, but based on a very lower number of tested individuals (*n* = 26) and a very broad 95% CI (0.0–25.0).

Other frequently patch tested DKG series in WW with OD were the series for “resins/adhesives” (*n* = 295, 62.6%), “ingredients of topical preparations” (*n* = 157, 54.6%), “preservatives” (*n* = 257, 54.6%), and “rubber” (*n* = 203, 43.1%). As shown in Table 5, the most frequent sensitisers in the DKG series “resins/adhesives” were 4,4′‐diaminodiphenylmethane (MDA, 7.0%, 95% CI 4.3–10.6), 1,6‐hexanediol‐diglycidyl ether (5.0%, 95% CI 2.5–8.7), benzoyl peroxide (4.9%, 95% CI 2.7–8.1), 1,4‐butanediol‐diglycidyl ether (4.7%, 95% CI 2.3–8.4), and toluylene diisocyanate (3.7%, 95% CI 0.5–12.7). No sensitisations to any of the “ingredients of topical preparations” were particularly frequent (Table S3). Among the “preservatives”, only sensitisations to methyldibromoglutaro nitrile 0.3% pet. (MDBGN, 4.3%, 95% CI 1.2–10.5) and methylisothiazolinone (MI, 3.9%, 95% CI 1.7–7.6) were frequent when these allergens were tested as part of this special series (Table S4). In the “rubber” series, only the positive patch test reactions to 1,3‐diphenylguanidine (1,3‐DPG, 5.5%, 95% CI 2.8–9.6) stood out (Table S5).

**TABLE 5:** Reaction frequencies, supplemented with 95% confidence intervals (95% CI), to allergens of the DKG series “resins/adhesives” in woodworkers with occupational dermatitis.

Substance	Conc.	Tested *n*	Pos. *n*	Raw pos. % [95% CI]
4,4′‐Diaminodiphenylmethane	0.5%	287	20	7.0 [4.3–10.6]
1,6‐Hexanediol‐diglycidyl ether	0.25%	221	11	5.0 [2.5–8.7]
Benzoyl peroxide	1%	286	14	4.9 [2.7–8.1]
1,4‐Butanediol‐diglycidyl ether	0.25%	214	10	4.7 [2.3–8.4]
Toluylene diisocyanate	1%	54	2	3.7 [0.5–12.7]
Phenylglycidyl ether	0.25%	280	7	2.5 [1.0–5.1]
Diethylene triamine	1%	222	5	2.3 [0.7–5.2]
Ethyl‐2‐cyanacrylat	10%	43	1	2.3 [0.1–12.3]
Isophorondiamine (IPD)	0.5%	286	6	2.1 [0.8–4.5]
Butyl glycidyl ether	0.25%	287	4	1.4 [0.4–3.5]
P.‐tert.‐butylphenyl glycidyl ether	0.25%	158	2	1.3 [0.2–4.5]
Phenol formaldehyde resin (Novolak)	5%	280	3	1.1 [0.2–3.1]
Hydroxyethyl acrylate	0.1%	285	3	1.1 [0.2–3.0]
Triethyleneglycol dimethacrylate (TEGDMA)	2%	286	3	1.0 [0.2–3.0]
BIS‐GMA	2%	287	3	1.0 [0.2–3.0]
M‐xylidendiamine	0.1%	198	2	1.0 [0.1–3.6]
Hydroquinone	1%	115	1	0.9 [0.0–4.7]
Cresylglycidyl ether	0.25%	262	2	0.8 [0.1–2.7]
Ethylenglycol dimethacrylate	2%	286	2	0.7 [0.1–2.5]
2‐Hydroxyethyl methacrylae (HEMA)	1%	286	2	0.7 [0.1–2.5]
2‐Hydroxypropyl methacrylate (HPMA)	2%	288	2	0.7 [0.1–2.5]
Trimethylhexane‐1,6‐diamine	0.5%	191	1	0.5 [0.0–2.9]
Trimethylolpropane triglycidyl ether	0.25%	192	1	0.5 [0.0–2.9]
p‐tert.‐Butylcatechol	0.25%	258	1	0.4 [0.0–2.1]
Isobornyl acrylate	0.1%	55	0	0.0 [0.0–6.5]
Diphenylmethane‐4,4′‐diisocyanate	1%	56	0	0.0 [0.0–6.4]
Phenol formaldehyde resin (Resol)	5%	59	0	0.0 [0.0–6.1]
Diethylene triamine	0.5%	66	0	0.0 [0.0–5.4]
2,4,6‐Tris(dimethylaminomethyl)phenol	0.5%	77	0	0.0 [0.0–4.7]
4,4′‐Dihydroxydiphenyle	0.1%	179	0	0.0 [0.0–2.0]
Methylmethacrylate (MMA)	2%	286	0	0.0 [0.0–1.3]

*Note*: Vehicle is petrolatum unless aqua (aq.) is specified.

Abbreviations: 95% CI, 95% confidence interval; aq, aqua; conc., concentration; DKG, German Contact Dermatitis Research Group; pos., positive.

Among the 153 WW with OD and allergic contact dermatitis as the main diagnosis, the most common sensitisations were those to epoxy resin (*n* = 22), colophonium (*n* = 16), 4,4′‐diaminodiphenylmethane (*n* = 14), *Myroxylon pereirae* (*n* = 14), and propolis (*n* = 13) (Table S6). Of these, 21 (13.7%) had face dermatitis and the most frequent sensitisations in this subgroup were the same complemented with 1,6‐hexanediol diglycidyl ether, which was unfortunately only tested in 12/21 (57.1%) of the WW with OD (Table S7).

One hundred WW with OD were simultaneously patch tested with PPD and MDA. Two of the 5 patients with sensitisation to PPD had a concomitant positive reaction to MDA (40.0%), whereas 2 of the 7 patients with sensitisation to MDA (22.2%) had a positive reaction to PPD. Fifty‐three WW with OD were simultaneously patch tested with PPD and toluylene diisocyanate. Two had a positive reaction to each of them, but no concomitant reactions were found.

Three hundred five of 471 WW with OD were simultaneously patch tested with epoxy resin, colophonium, and 4,4′‐diaminodiphenylmethane. This group included 33 patients with face dermatitis. In a multivariate logistic regression analysis, face dermatitis was significantly associated with colophonium sensitisation (odds ratio [OR] 3.79 [95% CI 1.24–11.61]), whereas epoxy resin (OR 1.91 [95% CI 0.59–6.22]) and 4,4′‐diaminodiphenylmethane (OR 2.47 [0.76–8.03]) had a positive OR that was not significant.

## 4 Discussion

To the best of our knowledge, we here present the largest epidemiological study separately analysing contact sensitisations in WW with OD covering a period of 24 years. The data demonstrate that allergic contact dermatitis is common in WW with OD. The most important allergens include resins and plant‐derived substances.

### 4.1 Patient Characteristics

Similar to what has been reported for construction workers, among which WW are often included, we found a male predominance among WW with OD [4, 16]. On average, they were younger than non‐WW with OD working in other professions. This may indicate that OD occurs rather early in the professional career of WW, possibly related to a high amount of exposure to skin hazards, including strong allergens (e.g., epoxy resin). As in other professions, primarily the hands are exposed to these hazards [17]. Accordingly, WW with OD mainly suffered from hand dermatitis. Compared to non‐WW with OD, hand dermatitis was, however, significantly less common and instead, face dermatitis was significantly more common. A high share of face dermatitis in WW with OD has been reported before [4]. This suggests that airborne exposure to irritants and/or allergens is prevalent in this profession. In line with this, dusts were considered the main relevant co‐factor contributing to the dermatitis which was much more common among WW with OD than in non‐WW with OD. WW are highly exposed to wood dust (e.g., during sawing, grinding or filing of wood) and other dusts when finishing surfaces or working with insulating materials (e.g., fibreglass) which may cause airborne contact dermatitis in uncovered body sites, such as the face, the neck and lower arms [18]. Moreover, exposure to volatile allergens (e.g., epoxy resin) must be considered as 13.7% of patients with allergic contact dermatitis as the main diagnosis had face involvement. In contrast, the high share of face dermatitis in WW without OD is probably primarily related to leave‐on cosmetics which were the most frequent suspected allergen source in this group. Another important co‐factor in woodwork is mechanical strain, whereas compared to patients working in other professions with OD, wetness and occlusive environment were less frequently identified co‐factors in WW with OD, indicating rather dry working conditions and limited wearing of occlusive gloves.

Looking at the main diagnoses, allergic contact dermatitis was much more common than irritant contact dermatitis indicating that exposure to allergens is often the main cause of OD in woodwork. Irritant contact dermatitis was significantly less common than in non‐WW with OD suggesting that skin irritation is more prevalent in other occupations. Notably, even though much less frequent than allergic and irritant contact dermatitis, hyperkeratotic dermatitis was significantly more common among WW with OD than among WW without OD and non‐WW with OD. Hyperkeratotic hand dermatitis mainly affects the palms and is more frequent in men than in women [19]. A causal relationship between mechanical strain (e.g., friction, pressure) and the development or worsening of hyperkeratotic hand dermatitis has been suggested. As already mentioned, the hands of WW are subject to an increased degree of mechanical strain due to for example, handling of tools, carrying heavy objects, sawdust, splinters and rough timber surfaces, making the higher prevalence of hyperkeratotic dermatitis among WW with OD plausible. Atopic dermatitis is considered a risk factor for developing OD, particularly of irritant contact dermatitis [20]. Notably, atopic dermatitis was less frequent in WW with OD than in non‐WW with OD which may explain the lower prevalence of irritant contact dermatitis in the study group and might be the result of a healthy worker effect, that is, individuals with atopic dermatitis chose not to work in this profession or leave the profession because of work‐related skin problems.

### 4.2 Glues and Resins

Sensitisation to epoxy resin (diglycidyl ether of bisphenol A) was the only sensitisation within the DKG baseline series that was significantly more common among male WW with OD compared to male WW without OD and the most frequent sensitisation in WW with OD and allergic contact dermatitis as the main diagnosis. Epoxy resin is a constituent of epoxy resin systems which are used for example, as adhesives, protective coatings or binders in paints and varnishes [21, 22, 23]. Sensitisation to epoxy resin is usually work‐related and therefore, associated with OD in patch‐tested patients, especially in those working in craft occupations [5, 17]. Due to its abundant use and high sensitisation potential, sensitisations to epoxy resin have increased in the past years, particularly in construction workers [16, 24]. In accordance with our data, others also reported a high prevalence of sensitisation to epoxy resin in WW with OD, such as carpenters and joiners [4, 5, 25]. As it is used in many occupations, it was not unexpected that sensitisation to epoxy resin was similarly frequent in non‐WW with OD. Other constituents of epoxy resin systems are reactive diluents and hardeners, which are as well potent sensitisers [26, 27]. In line with this, WW with OD were also frequently sensitised to the reactive diluents 1,6‐hexanediol diglycidyl ether and 1,4‐butanediol diglycidyl ether. Here, apart from co‐exposure, immunological cross‐reactivity between each other and to epoxy resin must be considered [27]. Epoxy resin and other constituents of epoxy resin systems are volatile and well‐known causes of airborne allergic contact dermatitis in sensitised individuals [21, 23, 28]. Therefore, it is very likely that the high prevalence of face dermatitis in WW with OD is also related to the high prevalence of sensitisations and exposure to these allergens, even though the logistic regression analysis did not reveal a significant association between face dermatitis and epoxy resin sensitisation. In line with this, sensitisation to epoxy resin was most common in WW with OD and allergic contact dermatitis who had face involvement. The use of protective gloves and very diligent work hygiene are essential when working with epoxy resin systems [22]. However, complete protection from airborne exposure, including exposure as a bystander, is often not possible which results in a poor prognosis for job continuation in sensitised individuals exposed to constituents of epoxy resin systems and related face dermatitis [29].

The frequent sensitisation to 4,4′‐diaminodiphenylmethane (syn. 4,4′‐methylenedianiline, MDA) is partially explained by its use as a hardener in epoxy resin systems in the past, but MDA also serves as an intermediate in the synthesis of methylene diphenyl diisocyanate (MDI) [30, 31]. Therefore, a positive patch test to MDA is considered a marker for MDI sensitisation [31]. Both MDI and toluylene diisocyanate (TDI) are precursors for the production of polyurethanes (PU). Products containing isocyanates, including those which form PU, such as coatings, paints, varnishes, foams and adhesives, are widely used across nearly all construction sectors, including the woodworking industry [32, 33]. MDA is a para‐amino compound and therefore, immunological cross‐reactivity to PPD and other para‐amino compounds is possible [30, 34]. However, the current data do not show a clear association between sensitisation to MDA and to PPD, rendering primary sensitisation to PPD an unlikely explanation for the high frequency of MDA sensitisations among WW with OD. In line with this, it should be noted that the observed high prevalence of sensitisation to PPD in WW with OD was most probably a rather random effect considering the low number of individuals tested with PPD and the broad 95% CI including zero.

The frequent positivity to benzoyl peroxide among WW with OD could be related to its use as an initiator for radical polymerisation in the manufacturing of synthetic resins and adhesives [35]. However, benzoyl peroxide 1% pet. is considered a problematic test preparation as it is significantly associated with weak positive patch test reactions and co‐reactivity to sodium lauryl sulfate [36, 37], and therefore with a high risk of false‐positive, irritant patch test reactions. As in painters [21], the occupational relevance of positive patch test reactions to benzoyl peroxide is usually untraceable in WW.

### 4.3 Plant‐Based Substances

Colophonium (rosin) is a complex mixture of various conifer resin acids such as abietic, dehydroabietic and pimaric acid which mainly derives from pine wood (Pinus species) [38]. This probably explains the higher sensitisation frequency to colophonium among male WW with OD than in those without. This difference, however, was not significant which could be related to the abundant non‐occupational exposure to conifers for example, by gardening or the occurrence of colophonium in numerous other applications, for example, medical plasters, wound dressings or cosmetics [38]. Due to the high content of abietic acid, colophonium is also a relevant allergen in other occupations, such as metalworkers [39], explaining the similarly high frequency of sensitisation to colophonium in non‐WW with OD. The logistic regression analysis revealed a significant positive association between face dermatitis and sensitisation to colophonium in WW with OD. Airborne allergic contact dermatitis related to colophonium in sawdust has been reported before [18]. In addition to skin irritation by dusts and the high frequency of sensitisations to epoxy chemicals, this probably contributed to the increased frequency of face dermatitis in WW with OD.

The only significantly more frequent sensitisation to a DKG baseline series allergen in male WW with OD compared to male non‐WW with OD was to propolis which is a bee resin composed of more than 100 components including flavonoids, phenolic compounds/polyphenols, terpenes/terpenoids, steroids, alcohols, and aromatic acids [40]. Among them are various plant resins, including those derived from buds of trees commonly used by WW, such as poplar species. *Myroxylon pereirae* is the balsam obtained from the bark of the tree known as *Myroxylon balsamum var. pereirae* . It contains various tree resins and essential oils found in woods explaining the high sensitisation frequency to *Myroxylon pereirae* in WW [41, 42, 43]. Moreover, co‐reactivity among colophonium, *Myroxylon pereirae* and propolis is a well‐documented phenomenon that is attributed to common or chemically similar plant‐based allergenic constituents [41, 42, 43].

### 4.4 Biocides

Sensitisation frequencies to MI and MDBGN were higher in WW with OD and non‐WW with OD than in WW without OD, reaching, however, no significance. Because of its previous abundant use in cosmetics, sensitisation to MI was very frequent in the IVDK population during the study period, reaching a peak in 2013/14 [44]. Due to EU regulation limiting the use of MI in cosmetics, it has since then substantially decreased again. However, MI is still commonly used in industrial products [45] which has resulted in increasing associations between MI sensitisation and hand dermatitis and OD in the recent past [44]. Due to its broad occurrence in water‐based paints and varnishes, painters with OD have been particularly affected. Similarly, occupational exposures to MI in WW are likely predominantly related to paints, varnishes or glues. Positive patch test reactions to MDBGN have to be interpreted with caution as false positive reactions, especially if it is tested at 0.3% pet., have to be taken into account [46]. In line with this, positivity to MDBGN 0.2% pet. was much lower. Current exposures to MDBGN are difficult to establish and historical sensitisation must be considered.

### 4.5 Other Allergens

Sensitisations to chromium and cobalt were significantly less frequently observed in male WW with or without OD than in male non‐WW with OD, indicating that both allergens, which occur for example, in cement and leather [47, 48], are not typical sensitisers in WW. Interestingly, in 25 carpenters with occupational allergic contact dermatitis evaluated in the Finnish Institute of Occupational Health between 1976 and 1999, the most frequent contact sensitisers were chromium and epoxy resin [25]. Similarly, in a register from Northern Bavaria covering the years 1990 till 1999, sensitisation to chromium was frequently found in wood processors with OD [4]. Since the adoption of the EU directive 2003/53/EC aiming at limiting hexavalent chromium levels in cement, the number of sensitisations to potassium dichromate among construction workers has substantially declined in the past 20 years [16, 49]. Similarly, the amount of hexavalent chromium in leather articles coming into contact with the skin has been limited by the EU regulation 301/2014 [50]. The rather low frequency of sensitisation to chromium in male WW with OD in this sample might be the result of these regulations but may also indicate only limited exposure to cement and leather (e.g., leather gloves) in male WW with OD.

Similarly, sensitisation to thiuram mix was significantly less frequent in male WW with OD than in male non‐WW with OD and sensitisation to 1,3‐DPG was the most prevalent single sensitisation to a rubber chemical based on the results of the aimed testing of the DKG “rubber” series. However, patch test results with 1,3‐DPG 1% pet. have to be interpreted with caution as this test preparation is known to elicit doubtful or irritant reactions quite frequently [51]. Overall, the results are very similar to what has been reported for painters and suggest that WW use protective rubber gloves rather infrequently in comparison to other professions [21]. In line with this, the occlusive environment was rarely identified as a co‐factor in WW with OD.

## 5 Limitations

Even though the patch test data collection and analysis within the IVDK is a long‐standing, well‐established procedure, the retrospective, multicenter study design may have introduced inherent biases, including misclassification or selection biases. Similarly, missing data (e.g., exclusion of WW with unknown OD status) may have led to further biases. During the 24‐year study period, the composition of the test series as well as the concentrations of single preparations changed, resulting in varying numbers of patients tested with each single test allergen which may have compromised the analyses. We here focused on male WW to ensure comparability with the comparison groups. Therefore, no firm conclusions about female WW with OD can be drawn. No data on the clinical relevance of positive patch test results and accompanying clinical severity or course of dermatitis are available. Moreover, no results of patch testing patients' own materials (e.g., wood dust) were included. Particularly, allergic reactions to exotic woods, which are well‐known causes of contact allergies in some WW [52], are therefore not covered in this analysis.

## 6 Conclusions

We here confirm that resins and plant‐derived substances are among the most important contact allergens in WW. Thus, in WW with suspected OD, patch testing should particularly include the DKG baseline series and the DKG test series “resins and glues”. Moreover, patch testing patient's own material (e.g., wood dust) should be considered. The high share of WW with allergic contact dermatitis and face dermatitis hampers preventive efforts. Avoidance of relevant allergens by substitution is usually not possible and possibilities to effectively prevent airborne OD or dust exposure are limited in WW. However, risk awareness and diligent work hygiene, particularly when working with epoxy resin systems, should be promoted and accompanied by instructions on how to use personal protective equipment, including appropriate gloves to reduce exposure to allergens and mechanical strain. Based on our data, the use of rubber gloves in protection against for example, epoxy resin systems, paints and varnishes could potentially be improved. Moreover, masks or respirators should be used in addition to face shields, goggles or safety glasses during dust‐generating work activities (e.g., when sawing wood). In case of airborne contact dermatitis to components of epoxy resin systems, the ventilation systems should be checked and if possible improved. However, in these cases it might be necessary to spare affected individuals from working with epoxy resin systems and in surrounding areas.

## Conflicts of Interest

The IVDK, maintained by the IVDK e.V., of which S.S. is an employee, is sponsored by the chemical, cosmetic and fragrance industry (associations) as well as by public funds. For details, see http://ivdk.org/en/sponsors/. D.B. has received honoraria for lectures from Novartis and Pfizer. R.B. has received honoraria for lectures and participation in advisory boards from LEO Pharma. H.D. is an investigator, consultant and/or speaker for Almirall Hermal, Stallergenes, LEO Pharma, Sanofi‐Aventis and Novartis Pharma. N.W. has received honoraria for lectures and advisory boards from ALK‐Abelló, LEO Pharma, Novartis Pharma, Takeda, Blueprint, CSL‐Behring, Biocryst, Synlab, Nutricia and was or is involved in clinical trials of Novartis Pharma, Sanofi, Blueprint, and Allakos. She received study grants from Novartis Pharma and Allergopharma. The other authors declare no conflicts of interest.

## Supporting information


**Data S1:** cod70041‐sup‐0001‐Supinfo.docx.

## Data Availability

Sharing of original data is not possible due to legal data protection restrictions.

## References

[1] T. Kauppinen , R. Vincent , T. Liukkonen , et al., “Occupational Exposure to Inhalable Wood Dust in the Member States of the European Union,” Annals of Occupational Hygiene 50, no. 6 (2006): 549–561, 10.1093/annhyg/mel013. 16571638

[2] M. C. Häberle , Kanerva's Occupational Dermatology, 3rd ed., ed. S. M. John , J. Duus Johansen , T. Rustemeyer , P. Elsner , and H. I. Maibach (Springer Cham, 2020), 1809–1817.

[3] T. Estlander , R. Jolanki , K. Alanko , and L. Kanerva , “Occupational Allergic Contact Dermatitis Caused by Wood Dusts,” Contact Dermatitis 44, no. 4 (2001): 213–217, 10.1034/j.1600-0536.2001.044004213.x. 11260236

[4] M. Bock , A. Schmidt , T. Bruckner , and T. L. Diepgen , “Occupational Skin Disease in the Construction Industry,” British Journal of Dermatology 149, no. 6 (2003): 1165–1171, 10.1111/j.1365-2133.2003.05748.x. 14674893

[5] K. Aalto‐Korte , K. Koskela , and M. Pesonen , “Construction Workers' Skin Disorders in the Finnish Register of Occupational Diseases 2005‐2016,” Contact Dermatitis 83, no. 6 (2020): 437–441, 10.1111/cod.13648. 32608063

[6] A. S. Quaade , F. Alinaghi , J. B. Dietz , C. Y. Erichsen , and J. D. Johansen , “Chronic Hand Eczema: A Prevalent Disease in the General Population Associated With Reduced Quality of Life and Poor Overall Health Measures,” Contact Dermatitis 89, no. 6 (2023): 453–463, 10.1111/cod.14407. 37634937

[7] P. V. Chernyshov , S. M. John , L. Tomas‐Aragones , et al., “Quality of Life Measurement in Occupational Skin Diseases. Position Paper of the European Academy of Dermatology and Venereology Task Forces on Quality of Life and Patient Oriented Outcomes and Occupational Skin Disease,” Journal of the European Academy of Dermatology and Venereology 34, no. 9 (2020): 1924–1931, 10.1111/jdv.16742. 32662100

[8] T. L. Diepgen , R. Scheidt , E. Weisshaar , S. M. John , and K. Hieke , “Cost of Illness From Occupational Hand Eczema in Germany,” Contact Dermatitis 69, no. 2 (2013): 99–106. 23869729 10.1111/cod.12038

[9] J. B. Dietz , T. Menne , H. W. Meyer , et al., “Degree of Employment, Sick Leave, and Costs Following Notification of Occupational Contact Dermatitis‐A Register‐Based Study,” Contact Dermatitis 84, no. 4 (2021): 224–235, 10.1111/cod.13719. 33058169

[10] A. Schnuch , J. Geier , H. Lessmann , R. Arnold , and W. Uter , “Surveillance of Contact Allergies: Methods and Results of the Information Network of Departments of Dermatology (IVDK),” Allergy 67, no. 7 (2012): 847–857. 22563651 10.1111/j.1398-9995.2012.02834.x

[11] W. Uter , A. Schnuch , and O. Gefeller , “Guidelines for the Descriptive Presentation and Statistical Analysis of Contact Allergy Data,” Contact Dermatitis 51, no. 2 (2004): 47–56. 15373843 10.1111/j.0105-1873.2004.00406.x

[12] V. Mahler , A. Nast , A. Bauer , et al., “S3 Guidelines: Epicutaneous Patch Testing With Contact Allergens and Drugs ‐ Short Version, Part 1,” Journal der Deutschen Dermatologischen Gesellschaft 17, no. 10 (2019): 1076–1093, 10.1111/ddg.13956. 31631537

[13] V. Mahler , A. Nast , A. Bauer , et al., “S3 Guidelines: Epicutaneous Patch Testing With Contact Allergens and Drugs ‐ Short Version, Part 2,” Journal der Deutschen Dermatologischen Gesellschaft 17, no. 11 (2019): 1187–1207, 10.1111/ddg.13971. 31765083

[14] A. Schnuch , “PAFS: Population‐Adjusted Frequency of Sensitization. (I) Influence of Sex and Age,” Contact Dermatitis 34, no. 6 (1996): 377–382, 10.1111/j.1600-0536.1996.tb02236.x. 8879920

[15] A. Schnuch , J. Geier , W. Uter , et al., “National Rates and Regional Differences in Sensitization to Allergens of the Standard Series. Population‐Adjusted Frequencies of Sensitization (PAFS) in 40,000 Patients From a Multicenter Study (IVDK),” Contact Dermatitis 37, no. 5 (1997): 200–209, 10.1111/j.1600-0536.1997.tb02435.x. 9412746

[16] J. Geier , A. Krautheim , W. Uter , H. Lessmann , and A. Schnuch , “Occupational Contact Allergy in the Building Trade in Germany: Influence of Preventive Measures and Changing Exposure,” International Archives of Occupational and Environmental Health 84, no. 4 (2011): 403–411. 20865273 10.1007/s00420-010-0581-8

[17] A. Bauer , M. Pesonen , R. Brans , et al., “Occupational Contact Allergy: The European Perspective‐Analysis of Patch Test Data From ESSCA Between 2011 and 2020,” Contact Dermatitis 88, no. 4 (2023): 263–274, 10.1111/cod.14280. 36694979

[18] D. K. Cook and S. Freeman , “Allergic Contact Dermatitis to Multiple Sawdust Allergens,” Australasian Journal of Dermatology 38, no. 2 (1997): 77–79, 10.1111/j.1440-0960.1997.tb01111.x. 9159962

[19] J. van der Heiden , T. Agner , T. Rustemeyer , and K. K. B. Clemmensen , “Hyperkeratotic Hand Eczema Compared to Other Subgroups of Hand Eczema—A Retrospective Study With a Follow‐Up Questionnaire,” Contact Dermatitis 78, no. 3 (2018): 216–222, 10.1111/cod.12945. 29314088

[20] M. G. Schütte , S. J. Tamminga , G. J. de Groene , S. Kezic , and H. F. van der Molen , “Work‐Related and Personal Risk Factors for Occupational Contact Dermatitis: A Systematic Review of the Literature With meta‐Analysis,” Contact Dermatitis 88, no. 3 (2023): 171–187, 10.1111/cod.14253. 36444496 PMC10107890

[21] S. Schubert , A. Bauer , U. Hillen , T. Werfel , J. Geier , and R. Brans , “Occupational Contact Dermatitis in Painters and Varnishers: Data From the Information Network of Departments of Dermatology (IVDK), 2000 to 2019,” Contact Dermatitis 85, no. 5 (2021): 494–502, 10.1111/cod.13935. 34260080

[22] K. Suuronen , B. Back , K. Aalto‐Korte , et al., “Skin Exposure to Epoxy Chemicals in Construction Coating, Assessed by Observation, Interviews, and Measurements,” Contact Dermatitis 80, no. 1 (2019): 18–25, 10.1111/cod.13122. 30259537

[23] K. Aalto‐Korte , M. Pesonen , and K. Suuronen , “Occupational Allergic Contact Dermatitis Caused by Epoxy Chemicals: Occupations, Sensitizing Products, and Diagnosis,” Contact Dermatitis 73, no. 6 (2015): 336–342. 26230376 10.1111/cod.12445

[24] L. Radillo , F. Riosa , M. Mauro , A. B. Fortina , M. T. Corradin , and F. Larese Filon , “Contact Dermatitis in Construction Workers in Northeastern Italian Patch Test Database Between 1996 and 2016,” Dermatitis 32, no. 6 (2021): 381–387, 10.1097/DER.0000000000000552. 34807530

[25] L. Kanerva , T. Leino , and T. Estlander , “Occupational Allergic Contact Dermatitis in Carpenters,” Contact Dermatitis 45, no. 1 (2001): 61–62, 10.1034/j.1600-0536.2001.045001061.x. 11422285

[26] J. Geier , H. Lessmann , U. Hillen , C. Skudlik , and U. Jappe , “Sensitization to Reactive Diluents and Hardeners in Epoxy Resin Systems. IVDK Data 2002‐2011. Part I: Reaction Frequencies,” Contact Dermatitis 74, no. 2 (2016): 83–93, 10.1111/cod.12491. 26538018

[27] J. Geier , H. Lessmann , U. Hillen , C. Skudlik , and U. Jappe , “Sensitization to Reactive Diluents and Hardeners in Epoxy Resin Systems. IVDK Data 2002‐2011. Part II: Concomitant Reactions,” Contact Dermatitis 74, no. 2 (2016): 94–101, 10.1111/cod.12490. 26537833

[28] K. Breuer , W. Uter , and J. Geier , “Epidemiological Data on Airborne Contact Dermatitis—Results of the IVDK,” Contact Dermatitis 73, no. 4 (2015): 239–247. 26234324 10.1111/cod.12455

[29] T. K. Caroe , N. E. Ebbehoj , J. P. Bonde , and T. Agner , “Occupational Hand Eczema and/or Contact Urticaria: Factors Associated With Change of Profession or Not Remaining in the Workforce,” Contact Dermatitis 78, no. 1 (2018): 55–63. 29076572 10.1111/cod.12869

[30] A. B. Fortina , S. Piaserico , F. Larese , et al., “Diaminodiphenylmethane (DDM): Frequency of Sensitization, Clinical Relevance and Concomitant Positive Reactions,” Contact Dermatitis 44, no. 5 (2001): 283–288, 10.1034/j.1600-0536.2001.440506.x. 11298694

[31] K. Aalto‐Korte , M. Pesonen , S. Suomela , and K. Suuronen , “Nine Years of Patch Testing With Isocyanates in a Clinic of Occupational Dermatology,” Contact Dermatitis 91, no. 3 (2024): 212–221, 10.1111/cod.14621. 38956835

[32] D. Bello , C. A. Herrick , T. J. Smith , et al., “Skin Exposure to Isocyanates: Reasons for Concern,” Environmental Health Perspectives 115, no. 3 (2007): 328–335, 10.1289/ehp.9557. 17431479 PMC1849909

[33] T. Estlander , H. Keskinen , R. Jolanki , and L. Kanerva , “Occupational Dermatitis From Exposure to Polyurethane Chemicals,” Contact Dermatitis 27, no. 3 (1992): 161–165, 10.1111/j.1600-0536.1992.tb05246.x. 1451461

[34] W. Uter , H. Lessmann , J. Geier , et al., “The Spectrum of Allergic (Cross‐)sensitivity in Clinical Patch Testing With ‘para Amino’ Compounds,” Allergy 57, no. 4 (2002): 319–322, 10.1034/j.1398-9995.2002.1o3314.x. 11906362

[35] K. Egele , H. Drexler , M. Fartasch , et al., “Benzoyl Peroxide's Sensitisation Potential and Potency in Experimental Methods and Review of Contact Allergy and Allergic Contact Dermatitis,” Contact Dermatitis 92 (2025): 436–445, 10.1111/cod.14765. 39910957 PMC12055316

[36] H. M. Ockenfels , W. Uter , H. Lessmann , A. Schnuch , and J. Geier , “Patch Testing With Benzoyl Peroxide: Reaction Profile and Interpretation of Positive Patch Test Reactions,” Contact Dermatitis 61, no. 4 (2009): 209–216, 10.1111/j.1600-0536.2009.01603.x. 19825092

[37] J. Geier , W. Uter , C. Pirker , and P. J. Frosch , “Patch Testing With the Irritant Sodium Lauryl Sulfate (SLS) is Useful in Interpreting Weak Reactions to Contact Allergens as Allergic or Irritant,” Contact Dermatitis 48, no. 2 (2003): 99–107, 10.1034/j.1600-0536.2003.480209.x. 12694214

[38] A. M. Downs and J. E. Sansom , “Colophony Allergy: a Review,” Contact Dermatitis 41, no. 6 (1999): 305–310. 10617209 10.1111/j.1600-0536.1999.tb06178.x

[39] S. Schubert , J. Geier , C. Skudlik , et al., “Relevance of Contact Sensitizations in Occupational Dermatitis Patients With Special Focus on Patch Testing of Workplace Materials,” Contact Dermatitis 83, no. 6 (2020): 475–486, 10.1111/cod.13688. 32829502

[40] K. Piontek , S. Radonjic‐Hoesli , J. Grabbe , et al., “Comparison of Patch Testing Brazilian (Green) Propolis and Chinese (Poplar‐Type) Propolis: Clinical Epidemiological Study Using Data From the Information Network of Departments of Dermatology (IVDK),” Contact Dermatitis 92, no. 3 (2025): 209–216, 10.1111/cod.14701. 39367763

[41] G. S. A. Nyman , A. M. Gimenez‐Arnau , J. Grigaitiene , L. Malinauskiene , E. Paulsen , and L. Hagvall , “Patch Testing With Propolis of Different Geographical Origins in a Baseline Series,” Acta Dermato‐Venereologica 101, no. 11 (2021): adv00591, 10.2340/actadv.v101.423. 34664078 PMC9455323

[42] G. Kocabas , N. A. Ipenburg , A. de Groot , and T. Rustemeyer , “Results of Patch Testing propolis in the European Baseline Series: A 4‐Year Retrospective Study,” Contact Dermatitis 91, no. 5 (2024): 375–378, 10.1111/cod.14678. 39169523

[43] W. Uter , S. M. Wilkinson , O. Aerts , et al., “Patch Test Results With the European Baseline Series, 2019/20‐Joint European Results of the ESSCA and the EBS Working Groups of the ESCD, and the GEIDAC,” Contact Dermatitis 87, no. 4 (2022): 343–355, 10.1111/cod.14170. 35678309

[44] A. Schnuch , S. Schubert , H. Lessmann , et al., “The Methylisothiazolinone Epidemic goes Along With Changing Patients' Characteristics—After Cosmetics, Industrial Applications Are the Focus,” Contact Dermatitis 82, no. 2 (2020): 87–93, 10.1111/cod.13414. 31599977

[45] C. Liden and I. R. White , “Increasing Non‐Cosmetic Exposure and Sensitization to Isothiazolinones Require Action for Prevention: Review,” Contact Dermatitis 90, no. 5 (2024): 445–457, 10.1111/cod.14523. 38382085

[46] A. Schnuch , S. Schubert , and J. Geier , “Clinicians vs. Epidemiologists: Patch Testing With Methyldibromo Glutaronitrile as a Controversial Issue,” Journal of the European Academy of Dermatology and Venereology 33, no. 6 (2019): e242–e244, 10.1111/jdv.15505. 30770592

[47] K. Kridin , R. Bergman , M. Khamaisi , S. Zelber‐Sagi , and S. Weltfriend , “Cement‐Induced Chromate Occupational Allergic Contact Dermatitis,” Dermatitis 27, no. 4 (2016): 208–214, 10.1097/DER.0000000000000203. 27331340

[48] D. Hamann , C. R. Hamann , P. Kishi , T. Menne , J. D. Johansen , and J. P. Thyssen , “Leather Contains Cobalt and Poses a Risk of Allergic Contact Dermatitis: Cobalt Indicator Solution and X‐Ray Florescence Spectrometry as Screening Tests,” Dermatitis 27, no. 4 (2016): 202–207, 10.1097/DER.0000000000000200. 27427822

[49] S. J. Stocks , R. McNamee , S. Turner , M. Carder , and R. M. Agius , “Has European Union Legislation to Reduce Exposure to Chromate in Cement Been Effective in Reducing the Incidence of Allergic Contact Dermatitis Attributed to Chromate in the UK?,” Occupational and Environmental Medicine 69, no. 2 (2012): 150–152, 10.1136/oemed-2011-100220. 21849347

[50] F. Alinaghi , C. Zachariae , J. P. Thyssen , and J. D. Johansen , “Temporal Changes in Chromium Allergy in Denmark Between 2002 and 2017,” Contact Dermatitis 80, no. 3 (2019): 156–161, 10.1111/cod.13181. 30443954

[51] J. Geier , H. Lessmann , V. Mahler , U. Pohrt , W. Uter , and A. Schnuch , “Occupational Contact Allergy Caused by Rubber Gloves—Nothing Has Changed,” Contact Dermatitis 67, no. 3 (2012): 149–156. 22762249 10.1111/j.1600-0536.2012.02139.x

[52] J. O. Podjasek , R. H. Cook‐Norris , D. M. Richardson , L. A. Drage , and M. D. Davis , “Allergic Contact Dermatitis From Exotic Woods: Importance of Patch‐Testing With Patient‐Provided Samples,” Dermatitis 22, no. 2 (2011): E1–E6. 21504691

