# Supplementary material for: Epidemiological Comparative Study on Contact Sensitisations in Woodworkers With Occupational Dermatitis: Patch Test Data of the Information Network of Departments of Dermatology, 1999 to 2023
Source: Contact Dermatitis. 2025 Nov 12;94(2):125–36. doi: 10.1111/cod.70041 (PMC12793820; doi:10.1111/cod.70041)
Supplement: Supplementary file 1 — Data S1: cod70041‐sup‐0001‐Supinfo.docx. [file COD-94-125-s001.docx]

**Supplemental Tables**

**Supplemental Table S1:** Occupations of woodworkers (WW) without occupational dermatitis (OD)

| Occupation | n | % |
| --- | --- | --- |
| Carpenter (construction, furniture, coffin), joiner | 269 | 48.2 |
| Wood preparer, sawmill worker, veneer producer, etc. | 101 | 18.1 |
| Roofer, prefabricated wood assembler | 95 | 17.0 |
| Carpenter, woodworker, etc. (unspecified) | 31 | 5.6 |
| Wood shaper, wood turner, wood carver | 24 | 4.3 |
| Model carpenter, mold carpenter | 13 | 2.3 |
| Wood surface finisher, stainer, polisher, veneerer | 13 | 2.3 |
| Wooden goods maker (brushes, toys, etc.) | 8 | 1.4 |
| Basket and wickerwork maker | 3 | 0.5 |
| Wainwright, cooper | 1 | 0.2 |

**Supplemental Table S2:** Occupations of patients not working as woodworkers (non-WW) with occupational dermatitis

| Occupation | n | % |
| --- | --- | --- |
| Hairdresser | 2382 | 6.0 |
| Nurse | 2101 | 5.3 |
| Janitor/Cleaner | 1637 | 4.1 |
| Elderly Caregiver | 1478 | 3.7 |
| Nurse/Midwife | 1073 | 2.7 |
| Mechanic (other) | 1040 | 2.6 |
| Unemployed | 1001 | 2.5 |
| Cook (hot and cold dishes) | 902 | 2.3 |
| *Worker with unspecified job description* | *786* | *2.0* |
| Metalworker (n.a.) | 766 | 1.9 |
| Turner | 764 | 1.9 |
| *Other or unknown profession* | *692* | *1.7* |
| Dental Assistant | 645 | 1.6 |
| Painter/Varnisher | 628 | 1.6 |
| Retiree | 576 | 1.4 |
| Commercial Vehicle Mechanic/Mechatronics Technician | 489 | 1.2 |
| Office Specialist/Clerk/Administrative Worker | 455 | 1.1 |
| *Missing or incorrect Information* | *443* | *1.1* |
| Assembler (other) | 426 | 1.1 |
| Salesperson (n.a.) | 421 | 1.1 |
| Milling Machine Operator | 411 | 1.0 |
| Mason | 404 | 1.0 |
| Physiotherapist | 391 | 1.0 |
| Baker/Pastry Maker | 387 | 1.0 |
| Dental Technician | 360 | 0.9 |
| Kitchen Assistant | 348 | 0.9 |
| Medical Assistant/Receptionist (doctor’s office) | 342 | 0.9 |
| Machine Fitter | 329 | 0.8 |
| Masseur/Physiotherapist/Medical Swimming Instructor | 322 | 0.8 |
| Toolmaker/Mould Maker | 318 | 0.8 |
| Nursing Assistant | 312 | 0.8 |
| Warehouse/Shipping Worker, Newspaper Delivery Person | 288 | 0.7 |
| Machine Setter (n.a.) | 278 | 0.7 |
| Gardener/Landscape Worker | 271 | 0.7 |
| Florist/Flower Binder | 259 | 0.7 |
| Electrical Installer/Electrician | 259 | 0.7 |
| Operating Room Nurse | 258 | 0.6 |
| Machinist (other) | 249 | 0.6 |
| Technician (other) | 248 | 0.6 |
| Nursing Assistant/Paramedic | 247 | 0.6 |
| Student | 246 | 0.6 |
| Farmer | 243 | 0.6 |
| Bakery Salesperson | 235 | 0.6 |
| Housewife | 221 | 0.6 |
| Doctor (non-surgical) | 220 | 0.6 |
| Vehicle Operator (car, truck, bus, taxi) | 217 | 0.5 |
| Chemical Laboratory Technician | 215 | 0.5 |
| Metal Shaper (sawing, filing, etc.) | 214 | 0.5 |
| Kindergarten Teacher/Childcare Worker | 212 | 0.5 |
| Tiler | 204 | 0.5 |
| Doctor (primarily surgical) | 203 | 0.5 |
| Medical Laboratory Technician (MTA, etc.) | 187 | 0.5 |
| Plastics Processor | 187 | 0.5 |
| Beautician/Foot Care Specialist | 187 | 0.5 |
| Medical Laboratory Technician/MTA, Radiology Assistant (etc.) | 182 | 0.5 |
| Others | 11.600 | 29.2 |

**Supplemental Table S3:** Reaction frequencies, supplemented with 95% confidence intervals (95% CI), to allergens of the DKG series “ingredients of topical preparations” in woodworkers with occupational dermatitis.

| Substance | conc. | tested  n | pos.  n | raw pos.  % [95%-CI] |
| --- | --- | --- | --- | --- |
| Amerchol L-101 | 50 % | 240 | 4 | 1.7 [0.5 - 4.2] |
| Octyl gallate | 0.3 % | 254 | 4 | 1.6 [0.4 - 4.0] |
| Propylene glycol (aq.) | 20 % | 248 | 3 | 1.2 [0.3 - 3.5] |
| Tert.-butylhydroquinone | 1 % | 254 | 2 | 0.8 [0.1 - 2.8] |
| Cetostearyl alcohol | 20 % | 243 | 1 | 0.4 [0.0 - 2.3] |
| Cocamidopropyl betaine (aq.) | 1 % | 248 | 1 | 0.4 [0.0 - 2.2] |
| Triethanolamine (tea) (trolamine) | 2.5 % | 253 | 1 | 0.4 [0.0 - 2.2] |
| Cetearyl glucoside | 5 % | 54 | 0 | 0.0 [0.0 - 6.6] |
| Decyl glucoside | 5 % | 74 | 0 | 0.0 [0.0 - 4.9] |
| Lauryl polyglucose (Lauryl glucoside) | 3 % | 74 | 0 | 0.0 [0.0 - 4.9] |
| Polyethylene glycol-400 % | 100 % | 74 | 0 | 0.0 [0.0 - 4.9] |
| Ethylhexylglycerin | 5 % | 83 | 0 | 0.0 [0.0 - 4.3] |
| Hydroquinone | 1 % | 116 | 0 | 0.0 [0.0 - 3.1] |
| Polyethylene glycol ointment DAB 8 | 100 % | 171 | 0 | 0.0 [0.0 - 2.1] |
| Benzophenone 4 | 10 % | 222 | 0 | 0.0 [0.0 - 1.6] |
| Butylhydroxyanisole (BHA) | 2 % | 246 | 0 | 0.0 [0.0 - 1.5] |
| Butylhydroxytoluene (BHT) | 2 % | 249 | 0 | 0.0 [0.0 - 1.5] |
| Coconut diethanolamide | 0.5 % | 241 | 0 | 0.0 [0.0 - 1.5] |

Note: Vehicle is petrolatum unless aqua (aq.) is specified.

Abbreviations: 95% CI, 95% confidence interval; conc., concentration; DKG, German Contact Dermatitis Research Group; pos., positive

**Supplemental Table S4:** Reaction frequencies, supplemented with 95% confidence intervals (95% CI), to allergens of the DKG series “preservatives” in woodworkers with occupational dermatitis.

| Substance | conc. | tested  n | pos.  n | raw pos.  % [95%-CI] |
| --- | --- | --- | --- | --- |
| Methyldibromo glutaronitrile | 0.3 % | 94 | 4 | 4.3 [1.2 - 10.5] |
| Methylisothiazolinone (aq.) | 0.05 % | 203 | 8 | 3.9 [1.7 - 7.6] |
| Iodopropynylbutyl carbamate (IPBC) | 0.2 % | 242 | 4 | 1.7 [0.5 - 4.2] |
| Sodium disulfide | 1 % | 86 | 1 | 1.2 [0.0 - 6.3] |
| Quaternium 15 | 1 % | 217 | 2 | 0.9 [0.1 - 3.3] |
| Triclosan | 2 % | 252 | 2 | 0.8 [0.1 - 2.8] |
| Methyldibromo glutaronitrile | 0.2 % | 139 | 1 | 0.7 [0.0 - 3.9] |
| Sodium benzoate | 5 % | 167 | 1 | 0.6 [0.0 - 3.3] |
| Diazolidinyl urea | 2 % | 200 | 1 | 0.5 [0.0 - 2.8] |
| Paraben mix | 16 % | 245 | 1 | 0.4 [0.0 - 2.3] |
| Imidazolidinyl urea (germall 115) | 2 % | 253 | 1 | 0.4 [0.0 - 2.2] |
| Sorbic acid | 2 % | 252 | 1 | 0.4 [0.0 - 2.2] |
| DMDM hydantoin (aq.) | 2 % | 250 | 1 | 0.4 [0.0 - 2.2] |
| Diazolidinyl urea | 1 % | 53 | 0 | 0.0 [0.0 - 6.7] |
| Polyaminopropyl biguanide (aq.) | 2.5 % | 58 | 0 | 0.0 [0.0 - 6.2] |
| Sodium benzoate | 2 % | 81 | 0 | 0.0 [0.0 - 4.5] |
| Quaternium 15 | 2 % | 33 | 0 | 0.0 [0.0 - 10.6] |
| Benzyl alcohol | 1 % | 251 | 0 | 0.0 [0.0 - 1.5] |
| Bronopol | 0.5 % | 242 | 0 | 0.0 [0.0 - 1.5] |
| Chloroacetamide | 0.2% | 255 | 0 | 0.0 [0.0 - 1.4] |
| Chlorhexidine digluconate (aq.) | 0.5 % | 256 | 0 | 0.0 [0.0 - 1.4] |

Note: Vehicle is petrolatum unless aqua (aq.) is specified.

Abbreviations: 95% CI, 95% confidence interval; conc., concentration; DKG, German Contact Dermatitis Research Group; pos., positive

**Supplemental Table S5:** Reaction frequencies, supplemented with 95% confidence intervals (95% CI), to allergens of the DKG series “rubber” in woodworkers with occupational dermatitis.

| Substance | conc. | tested  n | pos.  n | raw pos.  % [95%-CI] |
| --- | --- | --- | --- | --- |
| 1,3-Diphenylguanidine (DPG) | 1 % | 200 | 11 | 5.5 [2.8 - 9.6] |
| Benzoyl peroxide | 1 % | 145 | 5 | 3.4 [1.1 - 7.9] |
| Mercaptobenzothiazole | 2 % | 172 | 4 | 2.3 [0.6 - 5.8] |
| Zinc diethyldithiocarbamate (ZDEC) | 1 % | 186 | 4 | 2.2 [0.6 - 5.4] |
| N-Isopropyl-N'-phenyl-p-phenylene diamine (IPPD) | 0.1 % | 189 | 4 | 2.1 [0.6 - 5.3] |
| Tetraethylthiuramdisulfide (Disulfiram)(TETD) | 0.25 % | 194 | 4 | 2.1 [0.6 - 5.2] |
| Tetramethylthiurammonosulfide (TMTM) | 0.25 % | 192 | 3 | 1.6 [0.3 - 4.5] |
| Sodium disulfide | 1 % | 62 | 1 | 1.6 [0.0 - 8.7] |
| Morpholinylmercaptobenzothiazole | 0.5 % | 196 | 3 | 1.5 [0.3 - 4.4] |
| N-cyclohexyl-2-benzothiazyl sulfenamide | 1 % | 198 | 3 | 1.5 [0.3 - 4.4] |
| Tetramethylthiuramdisulfide (TMTD) | 0.25 % | 191 | 2 | 1.0 [0.1 - 3.7] |
| Ethylene diamine-di-HCl | 1 % | 200 | 2 | 1.0 [0.1 - 3.6] |
| Dibenzothiazyldisulfide (MBTS) | 1 % | 200 | 2 | 1.0 [0.1 - 3.6] |
| p-tert.-Butylcatechol | 0.25 % | 183 | 1 | 0.5 [0.0 - 3.0] |
| Methenamine (hexamethylene tetramine) | 1 % | 193 | 1 | 0.5 [0.0 - 2.9] |
| Dipentamethylen thiuramdisulfide | 0.25 % | 197 | 1 | 0.5 [0.0 - 2.8] |
| Monobenzone | 1 % | 199 | 1 | 0.5 [0.0 - 2.8] |
| N,N'-diphenyl-p-phenylene diamine (DPPD) | 0.25 % | 196 | 1 | 0.5 [0.0 - 2.8] |
| Zinc dibutyldithiocarbamate (ZDBC) | 1 % | 199 | 1 | 0.5 [0.0 - 2.8] |
| 4,4'-Dihydroxydiphenyle | 0.1 % | 171 | 0 | 0.0 [0.0 - 2.1] |
| Cyclohexyl thiophthalimide | 0.5 % | 195 | 0 | 0.0 [0.0 - 1.9] |
| Dibutyl thiourea | 1 % | 199 | 0 | 0.0 [0.0 - 1.8] |
| Diphenyl thiourea | 1 % | 199 | 0 | 0.0 [0.0 - 1.8] |

Note: Vehicle is petrolatum unless aqua (aq.) is specified.

Abbreviations: 95% CI, 95% confidence interval; conc., concentration; DKG, German Contact Dermatitis Research Group; pos., positive

| Substance | conc. | tested  n | pos.  n | raw pos.  % [95%-CI] | Share of all sensitizations to this hapten, n/n total† (%) |
| --- | --- | --- | --- | --- | --- |
| Epoxy resin | 1 % | 135 | 22 | 16.3 [10.5 - 23.6] | 22/25 (84.0) |
| Colophonium (Rosin) | 20 % | 136 | 16 | 11.8 [6.9 - 18.4] | 16/23 (69.6) |
| 4,4'-Diaminodiphenylmethane | 0.5 % | 111 | 14 | 12.6 [7.1 - 20.3] | 14/20 (70.0) |
| *Myroxylon pereirae* | 25 % | 136 | 14 | 10.3 [5.7 - 16.7] | 14/29 (48.2) |
| Propolis | 10 % | 136 | 13 | 9.6 [5.2 - 15.8] | 13/23 (56.5) |

**Supplemental Table S6:** Most frequent sensitizations, supplemented with 95% confidence intervals (95% CI), in woodworkers with occupational dermatitis and allergic contact dermatitis as main diagnosis (n=153)

†both sexes

Note: Vehicle is petrolatum.

Abbreviations: 95% CI, 95% confidence interval; conc., concentration; pos., positive

| Substance | conc. | tested  n | pos.  n | raw pos.  % [95%-CI] |
| --- | --- | --- | --- | --- |
| Epoxy resin | 1 % | 20 | 4 | 20.0 [5.7 - 43.7] |
| 4,4'-Diaminodiphenylmethane | 0.5 % | 16 | 3 | 18.8 [4.0 - 45.6] |
| Colophonium (Rosin) | 20 % | 20 | 3 | 15.0 [3.2 - 37.9] |
| *Myroxylon pereirae* | 25 % | 20 | 3 | 15.0 [3.2 - 37.9] |
| Propolis | 10 % | 20 | 3 | 15.0 [3.2 - 37.9] |
| 1,6-Hexanediol-diglycidyl ether | 0.25 % | 12 | 3 | 25.0 [5.5 - 57.2] |

**Supplemental Table S7:** Most frequent sensitizations, supplemented with 95% confidence intervals (95% CI), in woodworkers with occupational dermatitis and face dermatitis diagnosed with allergic contact dermatitis (n=21)

Note: Vehicle is petrolatum.

Abbreviations: 95% CI, 95% confidence interval; conc., concentration; pos., positive

**Acknowledgements**

The following IVDK centers have contributed data:

Aachen (J.M. Baron ), Aarau (J. Grabbe , K. Scherer-Hofmeier ), Augsburg (A. Ludwig , K. Siedlecki , S. Schuh ), Bad Reichenhall (K. Strom ), Basel (A. Bircher , K. Scherer , K. Hartmann ), Berlin B.-Frank. (B. Tebbe , R. Treudler ), Berlin BWK (A. Köhler ), Berlin Charité (T. Zuberbier , M. Worm ), Berlin-Neukölln (U. Hillen ), Bern (D. Simon ), Bielefeld (I. Effendy ), Bochum (Ch. Szliska , M. Straube , H. Dickel ), Bochum BGFA (M. Fartasch , M. Gina ), Bremen (D. Meyersburg , N. Patsinakidis ), Buxtehude (P. Große-Hüttmann , P. Hausenblas ), Dermatologikum (V. Martin , K. Reich , K. Breuer , D. Vieluf ), Dessau (A. Jung , U. Lippert ), Dortmund (P.J. Frosch , B. Mydlach , C. Pirker , R. Herbst , K. Kügler , U. Beiteke ), Dresden (G. Richter , R. Aschoff , P. Spornraft-Ragaller , A. Bauer ), Dresden Friedrichst. (A. Koch ), Erlangen (M. Fartasch , M. Hertl , V. Mahler , N. Wagner ), Essen (U. Hillen , J. Dissemond ), Falkenstein (H. Schwantes , D. Vieluf , M. Fischer , M. Gina ), Freudenberg (Ch. Szliska ), Geier Göttingen (J. Geier ), Gera (J. Meyer , H. Grunwald-Delitz , M. Kaatz ), Graz (W. Aberer , B. Kränke ), Greifswald (M. Jünger ), Göttingen (Th. Fuchs , J. Geier , T. Buhl ), Halle (G. Gaber , D. Lübbe , B. Kreft ), Hamburg (M. Kiehn , R. Weßbecher , E. Coors , J. Witte ), Hamburg BUK (K. Breuer , U. Seemann , C. Schröder-Kraft ), Hannover (T. Schaefer , Th. Werfel , A. Kapp , A. Heratizadeh ), Heidelberg (A. Schulze-Dirks , M. Hartmann , U. Jappe , M. Hartmann , K. Schäkel ), Heidelberg AKS (H. Dickel , T.L. Diepgen , E. Weisshaar ), Heilbronn (H. Löffler , P. Amann ), Homburg / Saar (F.A. Bahmer , P. Koch , C. Pföhler ), Jena (A. Bauer , W. Wigger-Alberti , M. Kaatz , S. Schliemann , S. Schliemann , J. Tittelbach , L. Ludriksone ), Kiel (J. Brasch , G. Heine ), Krefeld (A. Wallerand , M. Lilie , S. Wassilew ), Lausanne (P. Spring , C. Curdin ), Leipzig (R. Treudler , S. Forkel ), Linz (I. Angelova-Fischer ), Lippe Detmold (St. Nestoris ), Lübeck (J. Grabbe , I. Shimanovich , U. Jappe , K. Hartmann , À. Recke ), Mainz (D. Becker ), Mannheim (Ch. Bayerl , D. Booken , C.-D. Klemke , W. Ludwig-Peitsch , A. Schmieder , J. Nicolay ), Marburg (I. Effendy , H. Löffler , M. Hertl , W. Pfützner ), Minden (R. Stadler , J. Hoffmann , R. Gutzmer ), München LMU (T. Oppel , B. Przybilla , P. Thomas , T. Schuh , R. Eben , S. Molin , F. Rueff , E. Oppel , B. Summer ), München Schwabing (M. Agathos , K. Ramrath , M. Georgi , K. Ramrath , G. Isbary ), München TU (J. Rakoski , U. Darsow , T. Biedermann , K. Brockow ), Münster (B. Hellweg , R. Brehler , M. Behring , M. Sulk ), Nürnberg (I. Müller , D. Debus , A. Bachtler , K. Ertner , V. Baur ), Oldenburg (M. Padeken , O. Kautz , U. Raap , N. Patsinakidis ), Osnabrück (W. Uter , S.M. John , H.J. Schwanitz (+) , N. Schürer , H. Dickel , Ch. Skudlik , S.M. John , R. Brans ), Prager und Partner (V. Martin , W. Prager ), Rostock (H. Heise (+) , J. Trcka , S. Emmert , R. Panzer ), Stuttgart (J. Rieker-Schwienbacher ), Tobelbad (D. Wilfinger ), Tübingen (G. Lischka , M. Röcken , T. Biedermann , J. Fischer , S. Forchhammer , J. Fischer ), Ulm (H. Gall (+) , G. Staib , P. Gottlöber ), Ulm BWK (H. Pillekamp , D. Meine ), Ulm Univ.-Klinik (J. Weiss ), Wuppertal (O. Mainusch , J. Raguz ), Würzburg (J. Arnold , A. Trautmann ), Zwickau (B. Knopf , D. Teubner , D. Mechtel ), Zürich (B. Ballmer-Weber , A. Navarini , S. Micaletto , C. Lang )
